# Successful clearance of persistent SARS-CoV-2 asymptomatic infection following a single dose of Ad5-nCoV vaccine

**DOI:** 10.1038/s41392-023-01345-3

**Published:** 2023-03-15

**Authors:** Shaofu Qiu, Zhao Chen, Airu Zhu, Qiuhui Zeng, Hongbo Liu, Xiaoqing Liu, Feng Ye, Yingkang Jin, Jie Wu, Chaojie Yang, Qi Wang, Fangli Chen, Lan Chen, Sai Tian, Xinying Du, Qingtao Hu, Jinling Cheng, Canjie Chen, Fang Li, Jing Sun, Yanqun Wang, Jingxian Zhao, Jincun Zhao, Hongbin Song

**Affiliations:** 1 The Chinese PLA Center for Disease Control and Prevention, 100071 Beijing, China; 2 State Key Laboratory of Respiratory Disease, National Clinical Research Centre for Respiratory Disease, Guangzhou Institute of Respiratory Health, the First Affiliated Hospital of Guangzhou Medical University, 510182 Guangzhou, Guangdong China; 3 Pediatric Pulmonary Department, Guangzhou Women and Children’s Medical Center, Guangzhou Medical University, 510623 Guangzhou, Guangdong China; 4 Guangdong Provincial Center for Disease Control and Prevention, 510399 Guangzhou, Guangdong China; 5 Guangzhou Laboratory, Bio-island, 510320 Guangzhou, Guangdong China; 6 Institute of Infectious Disease, Guangzhou Eighth People’s Hospital of Guangzhou Medical University, 510060 Guangzhou, Guangdong China; 7 Shanghai Institute for Advanced Immunochemical Studies, School of Life Science and Technology, ShanghaiTech University, 201210 Shanghai, China; 8 National Clinical Research Center for Infectious Disease, Shenzhen Third People’s Hospital; The Second Affiliated Hospital, School of Medicine, Southern University of Science and Technology, 518112 Shenzhen, Guangdong China

**Keywords:** Vaccines, Infectious diseases

## Abstract

Persistent asymptomatic (PA) SARS-CoV-2 infections have been identified. The immune responses in these patients are unclear, and the development of effective treatments for these patients is needed. Here, we report a cohort of 23 PA cases carrying viral RNA for up to 191 days. PA cases displayed low levels of inflammatory and interferon response, weak antibody response, diminished circulating follicular helper T cells (cTfh), and inadequate specific CD4^+^ and CD8^+^ T-cell responses during infection, which is distinct from symptomatic infections and resembling impaired immune activation. Administration of a single dose of Ad5-nCoV vaccine to 10 of these PA cases elicited rapid and robust antibody responses as well as coordinated B-cell and cTfh responses, resulting in successful viral clearance. Vaccine-induced antibodies were able to neutralize various variants of concern and persisted for over 6 months, indicating long-term protection. Therefore, our study provides an insight into the immune status of PA infections and highlights vaccination as a potential treatment for prolonged SARS-CoV-2 infections.

## Introduction

The devastating global pandemic of coronavirus disease 2019 (COVID-19) is caused by SARS-CoV-2.^1,2^ As of January 2023, over 752 million confirmed cases and more than 6.8 million deaths have been reported worldwide,^3^ underscoring the continued threat to public health. A wide spectrum of clinical manifestations can be seen in COVID-19 patients ranging from asymptomatic, mild-to-moderate to severe-to-critical illness with multi-organ dysfunction and even death.^4^

Increasing evidence has highlighted the prevalence of asymptomatic infections,^5,6^ accounting for ~40–45% of SARS-CoV-2 infections.^7–9^ Notably, asymptomatic patients can also spread the virus efficiently^4,10^; furthermore, the duration of SARS-CoV-2 viral shedding is generally 3–46 days.^11–13^ However, some asymptomatic infections may persist with a longer duration of viral shedding as reported in our previous study;^14^ this persistence increases the risk of viral spread and may result in negative mental and physical consequences in these individuals as well as over-consumption of surveillance resources.^15^ Moreover, persistent infections may drive viral evolution; hence, the emergence of variants of concern (VOCs).^16^ Therefore, understanding the immune status of these persistently asymptomatic (PA) patients and developing treatments for the acceleration of viral clearance are critical to ending the pandemic.

Vaccination has been shown to be an effective way to not only build immunity in SARS-CoV-2-naive individuals, but also boost antiviral immunity in recovered people.^17^ Neutralizing antibody (nAb) titers were higher in convalescent patients with COVID-19 who received a single dose of mRNA vaccine than in naive individuals who received a third dose of mRNA vaccine.^17–22^ In addition, antigen-specific CD4^+^ and CD8^+^ T-cell responses induced by prior infections were enhanced by a single dose of mRNA vaccine.^23^ However, whether vaccinations can augment SARS-CoV-2-specific immune responses and accelerate viral clearance in patients with SARS-CoV-2 infection is unclear.

Here, we report a cohort of 23 PA patients with SARS-CoV-2 infection with a median viral shedding period of >100 days. All cases were young adults without immunodeficiency, but exhibited impaired immune activation against SARS-CoV-2, as indicated by lower levels of inflammation, interferon responses, antibody responses, specific CD4^+^ and CD8^+^ T-cell responses, as well as circulating follicular helper T cells (cTfh). Ten of the 23 cases remained SARS-CoV-2 RNA-positive 18 weeks post infection and received a single dose of adenovirus type-5 vector-based COVID-19 vaccine (Ad5-nCoV, trade-named Convidecia) for therapeutic purposes. Rapid and robust antibody and coordinated B-cell and cTfh responses were induced by vaccination, resulting in successful viral clearance. Furthermore, vaccine-elicited antibodies exhibited neutralizing activities against various VOCs and persisted for over 6 months. Our study affords an insight into the immune status of PA SARS-CoV-2 infections. Our results may guide vaccination strategies for the prevention and treatment of prolonged infections.

## Results

### Demographic characteristics

**Fig. 1:**
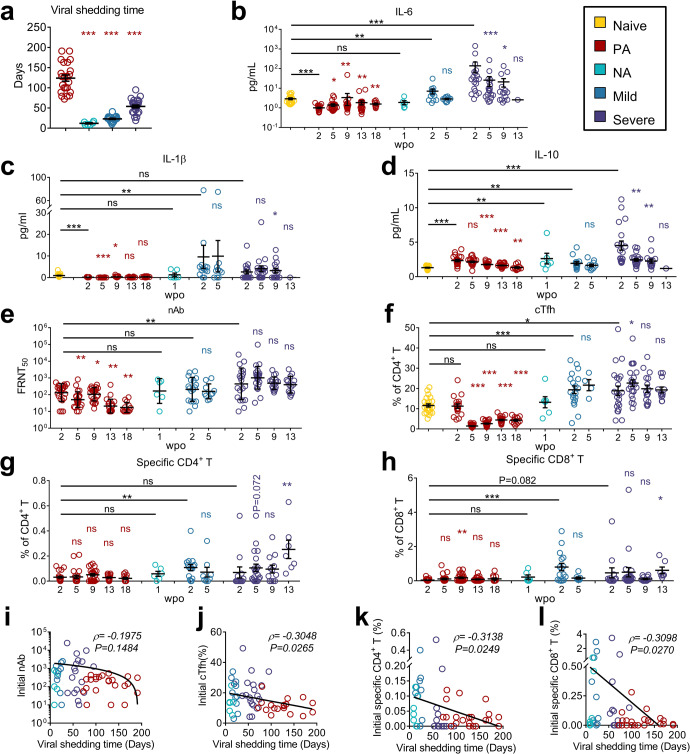
Persistent asymptomatic SARS-CoV-2-infected cases exhibited unique immunological features. **a** Viral shedding times are displayed. Comparisons between NA, Mild, and Severe groups with the PA group, respectively, were performed using Mann–Whitney test. **b**–**h** Plasma levels of IL-6 (**b**), IL-1β (**c**), IL-10 (**d**), nAb against SARS-CoV-2 (WT) (**e**), and frequencies of cTfh (**f**), specific CD4^+^ T (**g**), and specific CD8^+^ T (**h**) in PBMCs at various time points post-onset in each group are presented. wpo, weeks post-onset. Wilcoxon matched-pairs signed-rank test was used to compare the data of subsequent time points with the first time point in the same group. Mann–Whitney test was used to compare the data of the first time point in different groups with Naive or PA groups, as indicated. ns: *P* ≥ 0.05; *P < 0.05; **P < 0.01; ***P < 0.001. **i**–**l** Spearman correlation analysis was performed to analyze the correlation between viral shedding time and initial values of nAb (**i**), cTfh (**j**), specific CD4^+^ (**k**), and CD8^+^(**l**) T-cell frequencies, and the data of the four groups are presented. Data collected at the first time point for each patient was defined as the initial values. Each circle indicates an individual case, and the horizontal black lines indicate the mean ± SEM or geometric mean ± geometric SD (**e**) values. Naive, SARS-CoV-2-naive participants; PA, persistent asymptomatic SARS-CoV-2-infected cases; NA, non-persistent asymptomatic SARS-CoV-2-infected cases; Mild, patients with mild COVID-19; Severe, patients with severe COVID-19

Twenty-three patients with PA SARS-CoV-2 infection (PA group) were enrolled in this study; 6 patients with non-prolonged asymptomatic infection (NA group), 19 patients with mild COVID-19 (mild group), 25 patients with severe COVID-19 (severe group), and 20 SARS-CoV-2-naive donors (naive group) were included as controls. PA patients had no comorbidities, while some patients in other groups had various comorbidities (Supplementary Table 1). Patients were hospitalized or quarantined at the time of initial SARS-CoV-2 diagnosis until clearance of viral RNA. In general, the severe group exhibited longer viral shedding periods than the mild and NA groups (Supplementary Table 1). However, PA patients displayed the longest SARS-CoV-2 RNA shedding periods (Fig. 1a and Supplementary Table 1), with a median viral shedding time of 128 days. All patients were eventually cured.

### PA cases exhibited impaired immune activation

Cytokine storms are a hallmark of COVID-19.^24^ Inflammatory cytokines interleukin (IL)-6 and IL-1β levels were remarkably elevated in the mild and severe groups but not in PA cases (Fig. 1b, c). Compared to naive participants, all infected cases showed high initial levels of the regulatory cytokine IL-10; these levels decreased gradually (Fig. 1d). Similar to IL-6 and IL-1β, the lowest initial levels of IFN-α and IFN-λ, but not IFN-γ, were observed in PA cases (Supplementary Fig. 1a–c).

nAbs were induced at the early stage of SARS-CoV-2 infection and remained constant in the mild and severe groups (Fig. 1e). In PA cases, nAb titers at 2 weeks post infection onset (wpo) were lower than those in the NA group; however, this decrease did not reach statistical significance, possibly because of the limited numbers in the NA group (Fig. 1e). However, initial nAb titers were not directly correlated with viral shedding time (Fig. 1i). Despite prolonged viral shedding, the geometric mean titers of nAbs in the PA group decreased from 132 (95% CI: 78–224) at 2 wpo to 17 (95% CI: 11–26) at 18 wpo. Consistently, low abundance and declining kinetics of IgG and IgA antibodies against RBD, spike (S), S1, and S2 proteins were observed in the PA group (Supplementary Fig. 2a, b).

Corresponding to the limited antibody responses, both RBD-specific B cells and antibody-secreting cells (ASCs) remained in minor proportions during the infection (Supplementary Figs. 2c, d and 3). In addition, the cTfh population in PA cases diminished rapidly upon infection in contrast to the significant induction observed in the mild and severe groups (Fig. 1f). Overall, the initial frequency of cTfh was inversely associated with viral shedding time (Fig. 1j).

The antigen-specific T-cell response is another predominant component of antiviral immunity. Unlike the rapid and substantial induction in the mild group and the gradually increasing response in the severe group, both SARS-CoV-2-specific CD4^+^ and CD8^+^ T cells in PA cases remained low or undetectable (Fig. 1g, h). Specific CD4^+^ and CD8^+^ T cells were induced strongly early post infection in the mild group, moderately, but increased gradually, in the severe group, substantially and rapidly in NA cases, and weakly in PA cases from beginning to end (Fig. 1g, h). Combined analysis of data from patients and PA cases revealed that both initial frequencies of antigen-specific CD4^+^ and CD8^+^ T cells were negatively correlated with viral shedding time (Fig. 1k, l). To further characterize specific T-cell responses in PA cases during persistent SARS-CoV-2 infection, we evaluated their memory subset distribution. SARS-CoV-2-specific CD4^+^ T cells in PA cases predominantly comprised effector memory T cells (TEM, CCR7–CD45RA-) and central memory T cells (TCM, CCR7^+^CD45RA^-^) at an early stage. After 18 weeks of persistently low levels of viral burden, the proportion of both TEM and TCM decreased, while that of TEMRA increased (Supplementary Fig. 2e–g).

Overall, low inflammatory and interferon responses, limited induction of antibody responses, diminished cTfh, and inadequate specific CD4^+^ and CD8^+^ T-cell responses resembled impaired immune activation.

### In the PA group, delayed viral clearance was associated with initially lower IFN-λ and higher IL-10 levels

In the PA group, 13 cases had achieved viral clearance while the other 10 cases remained viral RNA-positive at 18 weeks post infection (Supplementary Fig. 4a, b). To further explore the potential reasons affecting the rate of viral clearance, PA cases were divided into two subgroups according to their viral shedding time (PA-1: viral shedding time <18 weeks; PA-2: viral shedding time >18 weeks) (Supplementary Fig. 5a). Higher initial viral loads and IL-10 levels, as well as lower IFN-λ levels, were observed in the PA-2 subgroup (Supplementary Fig. 5b–e). In addition, higher levels of IL-10 and lower levels of IFN-λ, were correlated with prolonged viral shedding time (Supplementary Fig. 5f, g). No difference in levels of IFN-α, IFN-γ, IL-1β, IL-6, or humoral or cellular responses was found between these two subgroups (Supplementary Fig. 5h–q).

### Single Ad5-nCoV vaccination resulted in robust antibody responses and SARS-CoV-2 clearance in the PA group

**Fig. 2:**
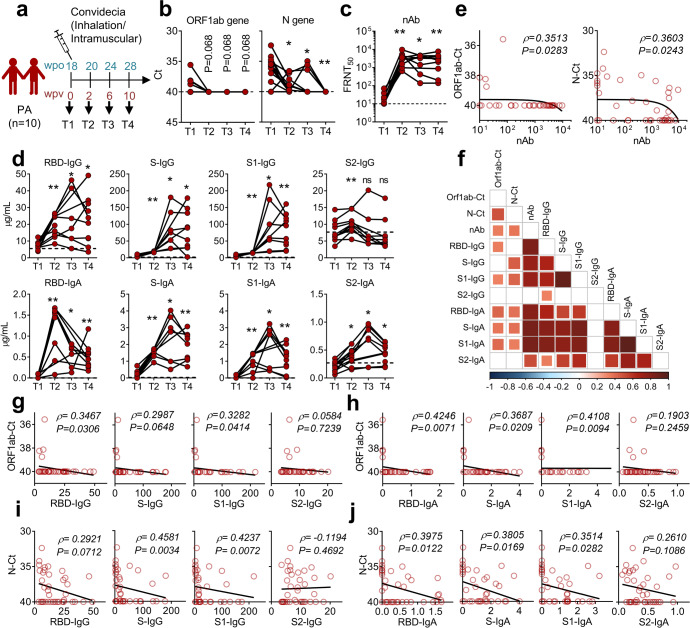
A single dose of Ad5-nCoV vaccine elicited robust antibody responses and viral clearance. **a** Vaccination scheme indicating that 10 of the PA cases received one dose of Ad5-nCoV vaccine (Convidecia*)* (9 via inhalation and 1 via intramuscular injection) at 18 wpo when they were still SARS-CoV-2 viral RNA-positive. Samples were collected at 4 consecutive time points (T1, T2, T3, and T4). **b** Viral loads in oropharyngeal swab were detected at given time points using q-RT-PCR targeting on *ORF1ab* (left) and *N* (right) genes. The dashed lines represent the threshold, and a Ct value of <40 indicates the presence of SARS-CoV-2 nucleic acid in the sample. **c** Dynamics of nAb titers against SARS-CoV-2 (WT) in PA cases post vaccination are presented. The dashed line represents the limit of detection. **d** Dynamics of IgG and IgA levels against multiple structural proteins (RBD, S, S1, and S2) in plasma are presented. Plasma samples from six healthy donors collected in 2017 − 2018 were used to define the thresholds (mean + 3 SD), indicated as dashed lines. **b**–**d** Wilcoxon matched-pairs signed-rank test was performed to compare data between T1 and other time points, respectively (*P < 0.05; **P < 0.01). **e**–**j** Spearman correlation analysis was performed to analyze the correlation between Ct values of *ORF1ab* or *N* genes and the values of neutralizing antibodies (**e**) or various binding antibodies (**g**–**j**), which are summarized in a heatmap (**f**)

To accelerate viral clearance, the 10 PA cases that remained viral RNA-positive at 18 weeks post infection received a dose of an adenovirus type-5 vector-based COVID-19 vaccine (Ad5-nCoV, trade-named Convidecia).^25^ Oropharyngeal swabs and peripheral blood samples were obtained at four key time points following vaccination: 0 (T1), 2 (T2), 6 (T3), and 10 (T4) weeks post vaccination (wpv), corresponding to 18, 20, 24, and 28 wpo, respectively (Fig. 2a). The dynamics of viral RNA and humoral and cellular immune responses were also monitored.

*ORF1ab* and *N* genes declined to undetectable levels within 2–10 weeks following Ad5-nCoV vaccination in PA patients (Fig. 2b, c). Rapid and universal serological responses were provoked in the 10 PA patients. SARS-CoV-2 nAb geometric mean titers increased from 17 (FRNT50 95% CI: 11–26) pre-vaccination to 2454 (95% CI: 1313–4589) at 2 wpv, with a 151-fold increase in geometric mean, and remained at 1542 (95% CI: 525–4532) at 10 wpv (Fig. 2c). Specific IgG and IgA levels against RBD, S, S1, and S2 proteins also increased upon vaccination (Fig. 2d). Ct values of *ORF1ab* and *N* genes were strongly positively correlated with antibody titers, including nAb (Fig. 2e), IgG, and IgA specific to SARS-CoV-2 RBD, S1, and S proteins (Fig. 2f–j). These findings suggest that the rapid and potent antibody responses induced by vaccination contributed to SARS-CoV-2 clearance in persistent infections.

### Coordinated B-cell and cTfh responses contributed to effective antibody responses toward vaccination in PA cases

**Fig. 3:**
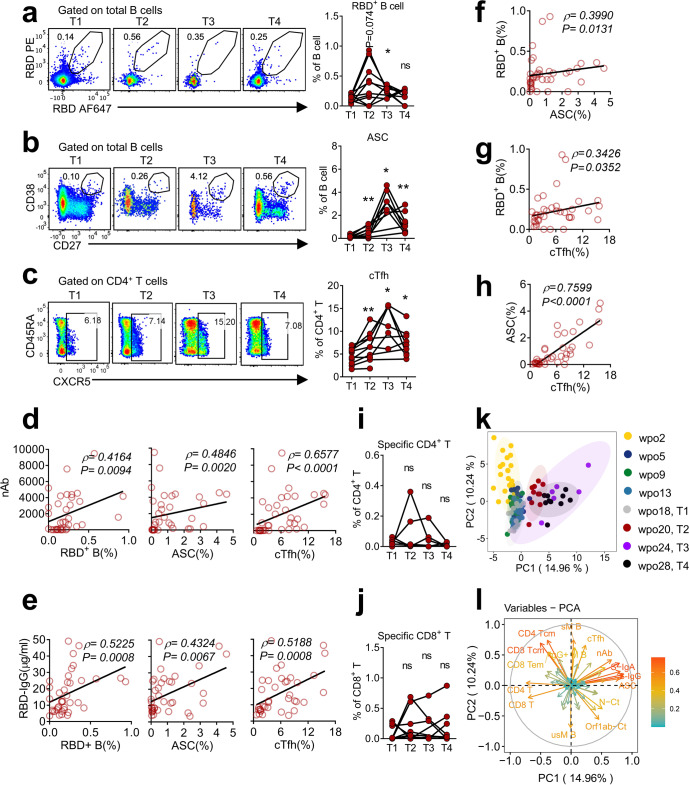
Ad5-nCoV vaccination enhanced B-cell and cTfh responses in PA cases; these responses were positively correlated with nAbs. **a**–**c** Frequencies of RBD^+^ B cells (**a**), ASCs (**b**), and cTfhs (**c**) in PBMCs of PA cases post vaccination are presented. Representative flow plots displaying the gating of RBD^+^ B cells (**a**), ASCs (**b**) and cTfhs (**c**) are presented on the left, and summary panels are presented on the right. **d**–**h** Spearman correlation analysis was performed to analyze the correlations among nAbs, RBD-IgG, RBD^+^ B cells, ASCs and cTfhs. **i**, **j** Frequencies of specific CD4^+^ (**i**) and CD8^+^ (**j**) T cells in PA cases post vaccination are presented. **k** PCA plot showing the distribution of patients in the PA group at different time points. Each dot represents a case. **l** PCA plot displaying corresponding trajectories of key markers affecting separation between groups. **a**–**c**, **i**, **j** Wilcoxon matched-pairs signed-rank test was conducted to compare data between T1 and other time points, respectively

Given the essential roles of B and cTfh cells in antibody production, various B-cell subsets and cTfh populations were further explored post vaccination. In accordance with potent antibody responses, the frequency of SARS-CoV-2 RBD^+^ B cells and ASCs rapidly increased and peaked at 2 and 6 wpv, respectively (Fig. 3a, b). Furthermore, cTfh cells exhibited similar dynamics to ASCs (Fig. 3c). Both nAb and RBD-specific IgG titers were positively correlated with RBD^+^ B cells, ASCs, and cTfhs (Fig. 3d, e). Moreover, all three populations were strongly positively correlated with each other (Fig. 3f–h).

In contrast to the vigorously boosted antibody responses, SARS-CoV-2-specific CD4^+^ and CD8^+^ T cells did not increase substantially post vaccination (Fig. 3i, j). Integrated analysis revealed that coordinated humoral and cTfh responses contributed most to the distinct immune trajectories towards Ad5-nCoV vaccination in PA cases (Fig. 3k, l). Collectively, these data demonstrated that humoral immune responses were potently enhanced following Ad5-nCoV vaccination, while antigen-specific CD4^+^ and CD8^+^ T-cell responses were modestly improved, indicating the predominant roles of enhanced humoral responses upon prime vaccination in clearing viral RNA and treating persistent SARS-CoV-2 infection.

### Single-vaccination dose induced durable and broad nAbs against various SARS-CoV-2 VOCs in PA cases

**Fig. 4:**
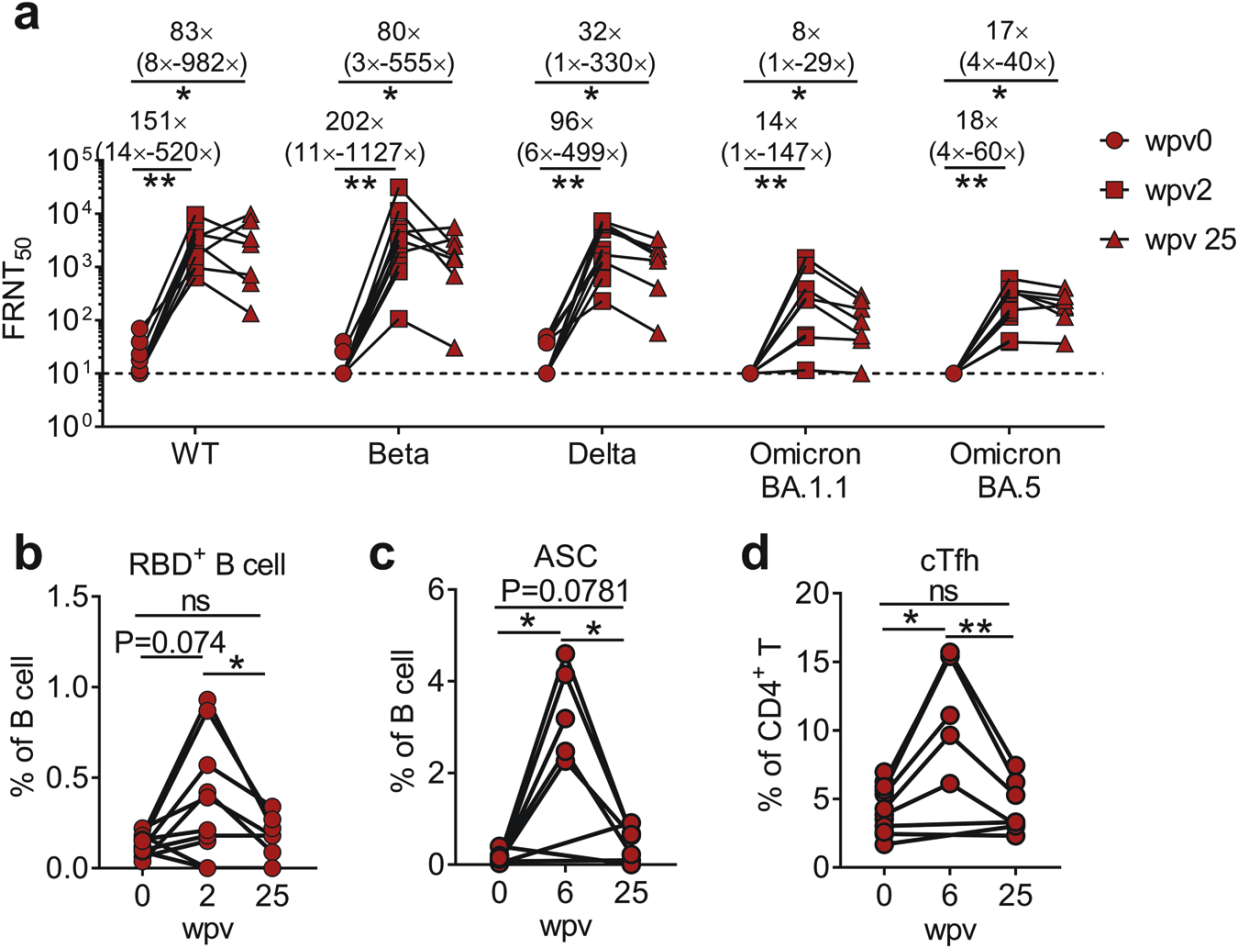
A single-vaccination dose induced durable and broad nAbs against various SARS-CoV-2 VOCs in PA cases. **a** nAb titers against five SARS-CoV-2 VOCs (WT, Beta, Delta, Omicron BA.1, and Omicron BA.5) at 0 wpv (before vaccination), 2 wpv (peak response) and 25 wpv are presented. The fold changes of nAb titers are presented as the geometric mean (min-max). **b**–**d** Frequencies of RBD^+^ B cells (**b**), ASCs (**c**), and cTfhs (**d**) at 0, 2, and 25 wpv are presented. Data were analyzed using Wilcoxon matched-pair signed-rank test

The ongoing transmission of SARS-CoV-2 has led to the emergence of VOCs with higher transmissibility or resistance to prior immunity.^26,27^ To further investigate the protection efficiency of this vaccine in facing emerging variants in PA cases, we evaluated neutralizing activity against the wild-type (WT) strain, B.1.351 (Beta), B.1.617 (Delta), BA.1.1 (Omicron), and BA.5 (Omicron) variants of SARS-CoV-2. Sera obtained from PA cases exhibited similar low neutralizing activity against WT, Beta, and Delta variants, and had no neutralizing effect on the Omicron variant (BA.1.1 and BA.5) before vaccination (Fig. 4a). At 2 wpv, nAb geometric mean titers against WT, Beta, Delta, and BA.1.1 (Omicron) and BA.5 (Omicron) variants increased to 2454 (95% CI: 1313–4589), 2553 (95% CI: 843–7737), 2027 (95% CI: 891–4612), 140 (95% CI: 46–425), and 180 (95% CI: 91–354) with geometric mean fold changes of 151-, 202-, 96-, 14-, and 18-times, respectively (Fig. 4a).

The longevity of effective neutralizing activity is essential for long-term protection against infection. Nevertheless, nAbs induced by vaccines, including mRNA, inactivated virus, and adv-vaccines, decay to low levels in 3–6 months.^28–30^ We, therefore, assessed the durability of vaccine-elicited nAbs against various VOCs. We observed that nAb geometric mean titers were sustained at 1627 (95% CI: 384–6892), 1111 (95% CI: 226–5453), 932 (95% CI: 258–3367), 81 (95% CI: 27–239), and 167 (95% CI: 81–343) up to 25 weeks post vaccination, respectively (Fig. 4a). Of note, compared with the peak (2 wpv), at 25 wpv, titers of nAbs against BA.5 (Omicron), the currently dominant VOC, were only slightly attenuated. Specific B cells, ASCs, and cTfh populations were also induced shortly post vaccination, and at 25 wpv, they waned to a level comparable to that at 0 wpv (Fig. 4b–d).

### Ad5-nCoV vaccine elicited comparable levels of nAb in PA cases and prior-vaccinated HDs

**Fig. 5:**
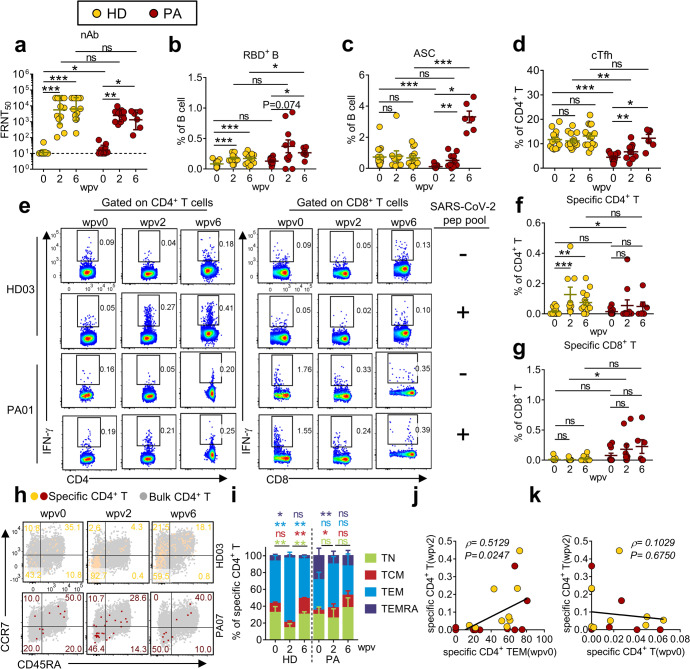
Ad5-nCoV vaccination induced distinct immune profiles in PA and HD groups. **a**–**g** Comparisons of nAbs against SARS-CoV-2 (WT) (**a**), RBD^+^ B cells (**b**), ASCs (**c**), cTfhs (**d**), specific CD4^+^ T cells (**f**), and specific CD8^+^ T cells (**g**) at 0, 2, and 6 wpv between PA and HD groups (SARS-CoV-2-naive participants who had completed two doses of inactivated vaccine) are presented. Representative flow plots of specific CD4^+^ and CD8^+^ T cells are presented (**e**). **f**, **g** The presented values were obtained with the formula: (unstimulated cells − (peptide-stimulated cells)). **h**, **i** Representative flow plots (**h**) and summary (**i**) of memory subsets of specific CD4^+^ T cells at 0, 2, and 6 wpv in the two groups are presented. Wilcoxon matched-pairs signed-rank test was performed to compare the data between different time points in the same group. **j**, **k** Spearman correlation test was conducted to analyze the correlation between specific CD4^+^ TEM (0 wpv) and specific CD4^+^ T (2 wpv) (**j**), and between specific CD4^+^ T before (wpv0) and after (wpv2) vaccination (**k**). nAb titers are presented as geometric mean ± geometric SD. Other data are presented as mean ± SEM

To further characterize the immune status elicited by a single Ad5-nCoV vaccine dose in PA cases, an HD group of SARS-CoV-2-naive participants who had completed a two-dose inactivated SARS-CoV-2 virus vaccine for 6 months (Supplementary Table 2) and received an additional boost with Ad5-nCoV vaccine inhalation was analyzed. The ages of the 10 PA cases ranged from 24 to 34 years (median, 27 years), and all were male. The ages of the 15 HD participants ranged from 22 to 44 years (median, 26 years), and 9 were female. Peripheral blood samples were collected at three key time points (pre-vaccination, 2, and 6 wpv); the kinetics of nAb titers against the vaccine variant (WT variant) and specific T- and B-cell responses were analyzed. Prior to receiving Ad5-nCoV vaccination, the geometric mean nAb titer was slightly higher in the PA group (17, 95% CI: 11–26) than in the HD group (11, 95% CI: 9–14) (Fig. 5a). At 2 wpv, nAb titers in both groups were augmented to a comparable level and were maintained for at least 6 weeks (Fig. 5a). Specific RBD^+^ B cells expanded in dynamics consistent with nAbs (Fig. 5b). While the frequency of ASCs increased significantly at 2 wpv and continued to increase to an average value of 3.204% in PA cases, no significant change was observed in HD recipients (Fig. 5c). The cTfh population exhibited disparate kinetics between PA and HD cases, similar to that of ASCs (Fig. 5d). The frequencies of ASCs and cTfh were significantly lower in PA cases than in HD participants before vaccination (Fig. 5c, d), indicating an impaired immune status in PA cases. Vaccine-primed cTfh cells in PA cases increased to a level equivalent to that in HD recipients. Moreover, ASCs reached a higher level in PA cases than in HD participants post vaccination (Fig. 5c, d), resembling a re-balanced immune status post vaccination in PA cases.

We then evaluated Ki67 expression to determine the proliferative capability of cTfhs. The proportion of cTfhs expressing Ki67 was significantly lower in PA cases than in HD participants before vaccination and approximated the proportion in HD participants at 2 wpv; this result could partially underpin the increase in cTfh frequency post vaccination (Supplementary Fig. 6a, b). Examination of total CD4^+^ T cells revealed that the proportion of Ki67-expressing cells in PA cases was comparable to that in HD participants before vaccination and was significantly higher than that in HD participants post vaccination (Supplementary Fig. 6a, c). This result indicates that low proliferative capacity was restricted to cTfhs but not to total CD4^+^ T cells in PA cases during persistent infection. Cytokine-skewed Tfh cells play critical roles in antibody class-switching and nAb generation, whereby the percentage of cTfh1 cells is broadly associated with antiviral humoral responses.^31^ Therefore, we assessed cytokine production in SARS-CoV-2-specific cTfhs. Ad5-nCoV vaccination resulted in cTfh1-polarization, as indicated by the production of IFN-g, TNF, and IL-2 in PA and HD groups (Supplementary Fig. 6d, e).

### Ad5-nCoV vaccine boosted antigen-specific CD4^+^ T-cell responses in HD recipients but not in PA cases

The magnitude of antigen-specific CD4^+^ T-cell responses increased significantly in HD recipients but not in PA cases post-Ad5-nCoV vaccination (Fig. 5e, f). In contrast, antigen-specific CD8^+^ T-cell responses did not exhibit significant changes in either HD or PA groups (Fig. 5e, g). We further analyzed the memory phenotype of antigen-specific CD4^+^ T cells. At 2 wpv, the TEM subset (CCR7^−^CD45RA^−^) was significantly increased in HD participants and exhibited a trend for an increase in PA cases, but this was not significant (Fig. 5h, i). No difference was observed in the memory subset composition of total CD4^+^ T cells between HD and PA groups pre- or post vaccination (Supplementary Fig. 6f). TEM cells are thought to circulate between peripheral and infection sites, and respond rapidly to re-stimulation.^32^ Here, we observed that vaccine-elicited specific CD4^+^ T-cell responses exhibited a strong positive correlation with pre-vaccination levels of specific CD4^+^ TEM cells but not with total specific CD4^+^ T cells (Fig. 5j, k).

## Discussion

Assessing the features and kinetics of immune responses is crucial for understanding the mechanisms underlying persistent infections. In this study, we describe the immune status of a cohort of 23 PA patients with COVID-19 who were all young adults without immunodeficiency disease or other comorbidities. Ten of these cases remained viral RNA-positive at 18 weeks post infection and received a dose of Ad5-nCoV vaccine (Convidecia) for therapeutic purposes.

Here, we found relatively low levels of inflammatory, interferon, antibody, and antigen-specific T-cell responses in PA cases, indicating that the inadequate induction of immune responses might contribute to the persistence of infection. In line with our findings, a number of studies identified the pivotal roles of specific antibodies and T-cell responses in viral clearance regarding MERS-CoV, SARS-CoV, and symptomatic SARS-CoV-2 infections.^33–36^ Within the PA group, delayed viral clearance correlated with elevated levels of anti-inflammatory IL-10 and reduced levels of antiviral IFN-λ. IL-10 suppresses adaptive immune responses and impairs the control and clearance of pathogens in multiple infection models,^37^ suggesting that the upregulation of IL-10 levels might contribute to the failure in viral clearance. However, the in-depth mechanism of how IL-10 regulates host antiviral responses in SARS-CoV-2 infections needs to be further dissected.

Convalescent patients with COVID-19 may benefit from vaccination whereby robust antibodies and specific T-cell responses can be elicited to protect the vaccinees against re-infection.^38^ However, whether vaccination could enhance immune responses in PA cases with an unresolved SARS-CoV-2 infection, helping to clear the virus or provide further protection against re-infection with various VOCs remained unclear. Here, we found that robust nAb responses were induced by vaccination in PA cases and strongly correlated with decreased viral loads; this result is consistent with the importance of nAb in viral control.^33^ Furthermore, these antibodies were able to persist at relatively high levels for over 6 months, whereas antibodies induced by two doses of mRNA or inactivated vaccines in SARS-CoV-2-naive participants were reported to decline at 6 months post vaccination.^28,39–41^ Notably, the nAbs could neutralize various SARS-CoV-2 VOCs circulating globally, including the currently dominant variant BA.5, implying protection against future emerging VOCs. One interesting question is why individuals who did not develop an appropriate immune reaction to the initial virus infection were capable to react robustly with a protective response to the vaccine. First, we speculate that this SARS-CoV-2 infection-vaccination scenario mimics the prime-boost immune process that could induce a robust immune response. Second, relatively low pro-inflammatory response in PA cases might be enhanced by the Ad5 vector and favor the induction of the immune response.^42–44^

SARS-CoV-2-naive participants who had completed two doses of inactivated vaccine benefited from an additional dose of Ad5-nCoV vaccine with an enhancement of antigen-specific CD4^+^ T-cell responses, consistent with the improved specific T-cell responses in those who received three doses of mRNA SARS-CoV-2 vaccines.^23,38^ However, one dose of Ad5-nCoV vaccine had minimal impact on the magnitude of antigen-specific CD4^+^ T-cell responses in PA cases. Antigen-specific CD4^+^ T-cell subsets in both HD and PA groups were predominantly TEM; additionally, higher levels of specific CD4^+^ TEM prior to vaccination were correlated with stronger specific CD4^+^ T-cell responses post vaccination. Thus, the minimal increase in antigen-specific CD4^+^ T cells upon vaccination in PA cases might be explained by the minor proportion of TEM and TCM and greater proportion of terminally differentiated CD4^+^ TEMRA prior to vaccination.

This study has several limitations. First, the present findings are based on a small cohort and a lack of parallel persistent infection queues that received other types of COVID-19 vaccines or those who received no vaccine. Whether other types of vaccines, such as mRNA and inactivated viral vaccines, would provide similar protection is unclear. Although one of the 10 PA cases received Ad5-nCoV vaccination via muscular injection and the others via inhalation, this participant’s immune response was not distinct from the others, indicating that the vaccine administration route did not affect the efficacy of vaccination in PA cases; however, this requires confirmation in a larger cohort. Second, although a 25-week follow-up assessment revealed durable protective antibody responses in PA cases post vaccination, accurate evaluation of the longevity of protectiveness requires an extended assessment period. Whether vaccinated PA cases would benefit from a booster dose of the vaccine warrants further investigation. Third, we did not address whether earlier vaccination could provide more effective and rapid protection in PA cases.

In summary, our study reveals that prolonged SARS-CoV-2 infection may be attributed to impaired immune activation, which manifested as low levels of initial inflammation, interferon, and weak antibody response, markedly reduced cTfh, as well as inadequate specific CD4^+^ and CD8^+^ T-cell responses. Ad5-nCoV vaccination resulted in rapid and robust antibody responses and coordinated B-cell and cTfh responses in PA cases, contributing to subsequent viral clearance. Furthermore, vaccination-elicited antibodies exhibited broad-spectrum neutralizing activity against various VOCs and long-lived activity. Our study of the immune status in persistent asymptomatic SARS-CoV-2 infections may facilitate subsequent exploration of the mechanisms underlying persistent infection. Importantly, we demonstrate for the first time that vaccination is an effective treatment for prolonged SARS-CoV-2 infection and propose the notion of a therapeutic vaccine for COVID-19 treatment.

## Materials and methods

### Patient enrollment and sample collection

A total of 23 patients with persistent asymptomatic (PA) SARS-CoV-2 infection confirmed by real-time reverse transcriptase-polymerase chain reaction (RT-PCR) were enrolled in this study. For comparison, 6 asymptomatic patients with non-persistent SARS-CoV-2 infection (NA), 19 mildly ill patients (MP), and 25 severely ill patients (SP) were also included in this study (Supplementary Table 1). All patients were infected with SARS-CoV-2 ancestry strain and were eventually cured. Nasopharyngeal swabs, plasma samples and peripheral blood mononuclear cells (PBMCs) were collected from each patient at multiple time points during their hospitalization. As asymptomatic infections do not result in the development of symptoms, the day of the first positive nucleic acid test was defined as day zero post-onset. In the PA group, 10 patients received a single dose of the COVID-19 vaccine (Convidecia) at 18 weeks post-onset (also termed week zero post vaccination) while still in the infectious phase (9 via inhalation and 1 intramuscularly). For comparison, this study included an HD group comprising SARS-CoV-2-naive participants who had finished a two-dose inactivated SARS-CoV-2 vaccine for 6 months and received an additional boost with Ad5-nCoV vaccine inhalation (Supplementary Table 2). Plasma samples from 19 SARS-CoV-2-naive donors collected in 2021–2022 were used as controls for cytokine assessment. PBMCs from 25 SARS-CoV-2-naive donors collected in 2021–2022 were used as controls for comparison before vaccination. Plasma samples from six healthy donors collected in 2017–2018 were used as controls for SARS-CoV-2-specific binding antibodies.

### Study approval

The study was approved by the Institutional Review Board of the First Affiliated Hospital of Guangzhou Medical University. Written informed consent was obtained from all participants. Patient samples were obtained at the indicated times, and clinical information was retrieved from clinical records.

### RT-PCR detection of SARS-CoV-2

Nucleic acids were extracted from nasopharyngeal swabs by using the Prefilled Viral Total NA Kit-Flex and KingFisher Flex System (Thermo Fisher Scientific Inc., Waltham, USA) according to the manufacturer’s protocol. The presence of SARS-CoV-2 was identified using an RT-PCR kit targeting the open reading frame 1ab (*ORF1ab*) and nucleocapsid protein (*N*) genes (Shanghai BioGerm Medical Biotechnology Co., Ltd., Shanghai, China). The results were considered positive when the cycle threshold (Ct) values of all genes were less than 40 cycles. Samples with Ct values of less than 40 cycles for only one gene were defined as inconclusive, and a second test was required.

### Cytokine measurements

The ELLA Simple Plex Cartridge Kit (Bio-Techne Co., Minnesota, USA) was used to measure cytokine levels according to the manufacturer’s instructions. The levels of interleukin 1 beta (IL-1β), interleukin-6 (IL-6), interleukin-10 (IL-10), and interferon-g (IFN-γ) were measured according to the manufacturer’s instructions. The levels of interferon-α (IFN-α) and interferon-λ (IFN-λ) were measured by using Human IFN-α ELISA KIT and Human IFN-λ ELISA KIT (COIBO BIO Inc., Shanghai, China) according to the manufacturer’s protocols.

### Focus reduction neutralization test

The SARS-CoV-2 focus reduction neutralization test (FRNT) was performed in a certified Biosafety Level 3 (BSL-3) laboratory. A volume of 50 μL of plasma samples was serially diluted, mixed with 50 μL of SARS-CoV-2 virus (100 focus forming units, FFU) in 96-well plates, and incubated for 1 h at 37 °C. The mixtures were then transferred to 96-well plates seeded with Vero E6 cells (ATCC, Manassas, VA) and incubated for 1 h at 37 °C to allow virus entry. The inocula were removed before adding the overlay media (100 mL MEM containing 1.2% carboxymethyl cellulose, CMC). The plates were then incubated at 37 °C for 24 h. The overlays were removed, and the cells were fixed with 4% paraformaldehyde solution for 30 min. Cells were permeabilized with 0.2% Triton X-100 and incubated with cross-reactive rabbit anti-SARS-CoV-N IgG (Catalog number: 40143-R001, Sino Biological, Inc., Beijing, China) for 1 h at 37 °C before adding HRP-conjugated goat anti-rabbit IgG (H^+^L) antibody (1:4000 dilution) (Catalog number: 111-035-144, Jackson ImmunoResearch, West Grove, PA, USA). The cells were incubated at 37 °C. The reactions were developed using KPL TrueBlue Peroxidase substrates (Seracare Life Sciences, Inc., Milford, MA, USA). The number of SARS-CoV-2 foci was calculated using an Elispot reader (Cellular Technology Ltd., Shaker Heights, OH). The SARS-CoV-2 strains used in this study were isolated from COVID-19 patients in Guangdong, China, including wild-type (SARS-CoV-2/human/CHN/IQTC01/2020, NCBI, accession number: MT123290), Beta (B.1.351), and Omicron (BA.1.1). The SARS-CoV-2 Delta (B.1.617.2, GDPCC) and Omicron (BA.5, GDPCC) variants were obtained from the Guangdong Provincial Centre for Disease Control and Prevention, China. Experiments related to authentic SARS-CoV-2 were conducted at the Guangzhou Customs District Technology Center Biosafety level 3 Laboratory.

### Detection of SARS-CoV-2-specific binding antibodies

Enzyme-linked immunosorbent assay (ELISA) was used to analyze SARS-CoV-2-specific IgG and IgA antibodies against the SARS-CoV-2 spike (S), spike RBD (RBD), spike 1 (S1), and spike 2 (S2) proteins with a commercial antibody detection kit provided by the R&D Department of AtaGenix Laboratories Co., Ltd., Wuhan, China. ELISA was performed on the plasma samples according to the manufacturer’s instructions. Plates were read using a BioTek Epoch microplate reader (BioTek Instruments, Inc., Vermont, USA) at an optical density (OD) of 450 nm. The standard curve of Ig concentration-OD450 was calculated using the standard substance in the kit that was detected simultaneously, and the Ig concentration in plasma was calculated.

### Peptide library

A set of 20-mer peptides encompassing four SARS-CoV-2 structures (S glycoprotein, and N, M and E proteins) and six putative accessory proteins (ORF3a [25396–26223], ORF6 [27205–27390], ORF7a [27397–27762], ORF8 [27897–28262], ORF9b [28287–28580], and ORF9c [28737–28958]) overlapping by 10 amino acids were synthesized and used to stimulate PBMCs. T-cell responses were measured using intracellular cytokine staining assays for interferon-γ (IFN-γ) and TNF.

### Detection of SARS-CoV-2-specific T cells

The following anti-human monoclonal antibodies were used: BB515-CD45RA (HI100), BUV737-CD45RA (clone: HI100), PerCP-Cy5.5-CD4 (clone: RPA-T4), BB515-CD4 (clone: RPA-T4), PerCP-Cy5.5-CD8 (clone: SK1), BV786-CD8 (clone: RPA-T8), BV650-CD56 (clone: NCAM16.2), BV510-CD3 (clone: HIT3a), BUV496-CD3 (clone: UCHT1), PE-Cy7-CCR7 (clone: G043H7), APC-R700-CXCR5 (clone: RF8B2), BV711-CXCR5 (clone: J252D4), APC-IFN-γ (clone: B27), BV421-IFN-γ (clone: B27), PE-TNF (clone: MAb11), APC-Cy7-TNF (clone: MAb11), BV605-IL-2 (clone: MQ1-17H12), BV786-Ki-67 (clone: B56), PE-Granzyme B (clone: GB11), PE-Dazzle594-IL-4 (clone: MP4-25D2), ef660-IL-21 (clone: 3A3-N2), R718-IL17A (clone: N49-653), and PE-Cy5-IL-10 (clone: JES3-19F1). Antibodies were purchased from BD Biosciences, Thermo Fisher Scientific, or BioLegend. PBMCs were prepared from blood samples using Lympholyte-H (Cedarlane), according to the manufacturer’s instructions. The cells were stored in liquid nitrogen until further analysis. For surface staining, 10^5^–10^6^ cells were stained with the indicated antibodies at 4 °C, and then labeled with cell viability staining dye (FVS440UV, BD Biosciences) at room temperature (20–25 °C) in the dark. For in vitro intracellular cytokine staining, 10^5^–10^6^ cells were cultured in a well of 96-well round-bottom plates at 37 °C for 12 h in the presence of 40 µM SARS-CoV-2 peptide pool and brefeldin A (BD Biosciences, 1:1000 final). The cells were then labeled for cell surface markers, fixed/permeabilized with Cytofix/Cytoperm Solution (BD Biosciences), and stained with anti-intracellular cytokine/protein antibodies. All flow cytometric data were acquired on a BD FACSFortessa and analyzed using FlowJo software (BD Biosciences).

### Detection of SARS-CoV-2-specific B cells

Fluorescent SARS-CoV-2-specific RBD probes were prepared by combining biotinylated proteins with fluorescently labeled streptavidin (SA). RBD probes were prepared at a ratio of 4 M of trimer to 1 M of SA. Two RBD probes, one labeled with phycoerythrin (PE) and one labeled with Alexa Fluor (AF)-647, were used in this panel to increase the specificity of detection of SARS-CoV-2-specific B cells. Cryopreserved PBMCs were thawed at 37 °C and stained with a flow cytometry panel consisting of viability dye (FVS440UV), IgM FITC, CD3 PE-CF594 (UCHT1), CD27 PE-Cy7 (M-T271), IgG APC-H7 (G18-145), CD138 BV421 (MI15), CD19 BV510 (SJ25C1), IgD BV650 (IA6-2), and CD38 BV786 (HIT2) (all from BD Biosciences). Cells were first stained with a cocktail of RBD probes for 60 min at 4 °C, washed with PBS, stained with the remaining antibody panel, and incubated for 15 min at room temperature. The cells were then resuspended in 2% FBS/PBS after fixing with Cytofix (BD Biosciences) for 30 min at 4 °C. All flow data were acquired on a BD FACSFortessa and analyzed using FlowJo software (BD Biosciences).

### Principal component analysis

Analysis and visualization were performed using R version 4.3.0 and Rstudio. Data were normalized, and packages including “FactoMineR”, “factoextra”, and “ggplot2” were used.

### Statistical analysis

Statistical analyses were performed using GraphPad Prism software version 7.00. The Wilcoxon matched-pair signed-rank test was used to compare differences within the same group at different time points. The Mann–Whitney *U* test was used to compare the differences between groups. Spearman’s correlation was used to assess the relationship between different factors. A *P* value <0.05 was considered statistically significant.

## Supplementary information

Supplementary file-clean version

## Data Availability

All data generated or analyzed during this study are included in this published article and its supplementary information files.
