# Supplementary material for: Successful clearance of persistent SARS-CoV-2 asymptomatic infection following a single dose of Ad5-nCoV vaccine
Source: Signal Transduct Target Ther. 2023 Mar 15;8:123. doi: 10.1038/s41392-023-01345-3 (PMC10015148; doi:10.1038/s41392-023-01345-3)
Supplement: Supplementary file 1 — Supplementary file-clean version [file 41392_2023_1345_MOESM1_ESM.docx]

Supplementary Materials for

Successful clearance of persistent SARS-CoV-2 asymptomatic infection following a single dose of Ad5-nCoV vaccine (Convidecia)

Shaofu Qiu^1, #^, Zhao Chen^2, #^, Airu Zhu^2, #^, Qiuhui Zeng^2, #^, Hongbo Liu^1, #^, Xiaoqing Liu^2^, Feng Ye^2^, Yingkang Jin^3^, Jie Wu^4^, Chaojie Yang^1^, Qi Wang^1^, Fangli Chen^2^, Lan Chen^2^, Sai Tian^1^, Xinying Du^1^, Qingtao Hu^2^, Jinling Cheng^2^, Canjie Chen^2^, Fang Li^2^, Jing Sun^2^, Yanqun Wang^2^, Jingxian Zhao^2,6 *^, Jincun Zhao^2,5,6,7,8 *^, Hongbin Song^1, *^

^1^The Chinese PLA Center for Disease Control and Prevention, 100071, Beijing, China.

^2^State Key Laboratory of Respiratory Disease, National Clinical Research Centre for Respiratory Disease, Guangzhou Institute of Respiratory Health, the First Affiliated Hospital of Guangzhou Medical University, 510182, Guangzhou, Guangdong, China.

^3^Pediatric Pulmonary Department, Guangzhou Women and Children's Medical Center, Guangzhou Medical University, 510623, Guangzhou, Guangdong, China.

^4^Guangdong Provincial Center for Disease Control and Prevention, 510399, Guangzhou, Guangdong, China.

^5^Institute of Infectious Disease, Guangzhou Eighth People's Hospital of Guangzhou Medical University, 510060, Guangzhou, Guangdong, China.

^6^Guangzhou Laboratory, Bio-island, 510320, Guangzhou, Guangdong, China.

^7^Shanghai Institute for Advanced Immunochemical Studies, School of Life Science and Technology, ShanghaiTech University, 201210, Shanghai, China.

^8^National Clinical Research Center for Infectious Disease, Shenzhen Third People's Hospital; The Second Affiliated Hospital, School of Medicine, Southern University of Science and Technology, 518112, Shenzhen, Guangdong, China

^#^ These authors contributed equally to this work.

Correspondence to: zhaojingxian@gird.cn, zhaojincun@gird.cn, hongbinsong@263.net.

**This PDF file includes:**

Figures. S1 to S6

Tables S1 to S2


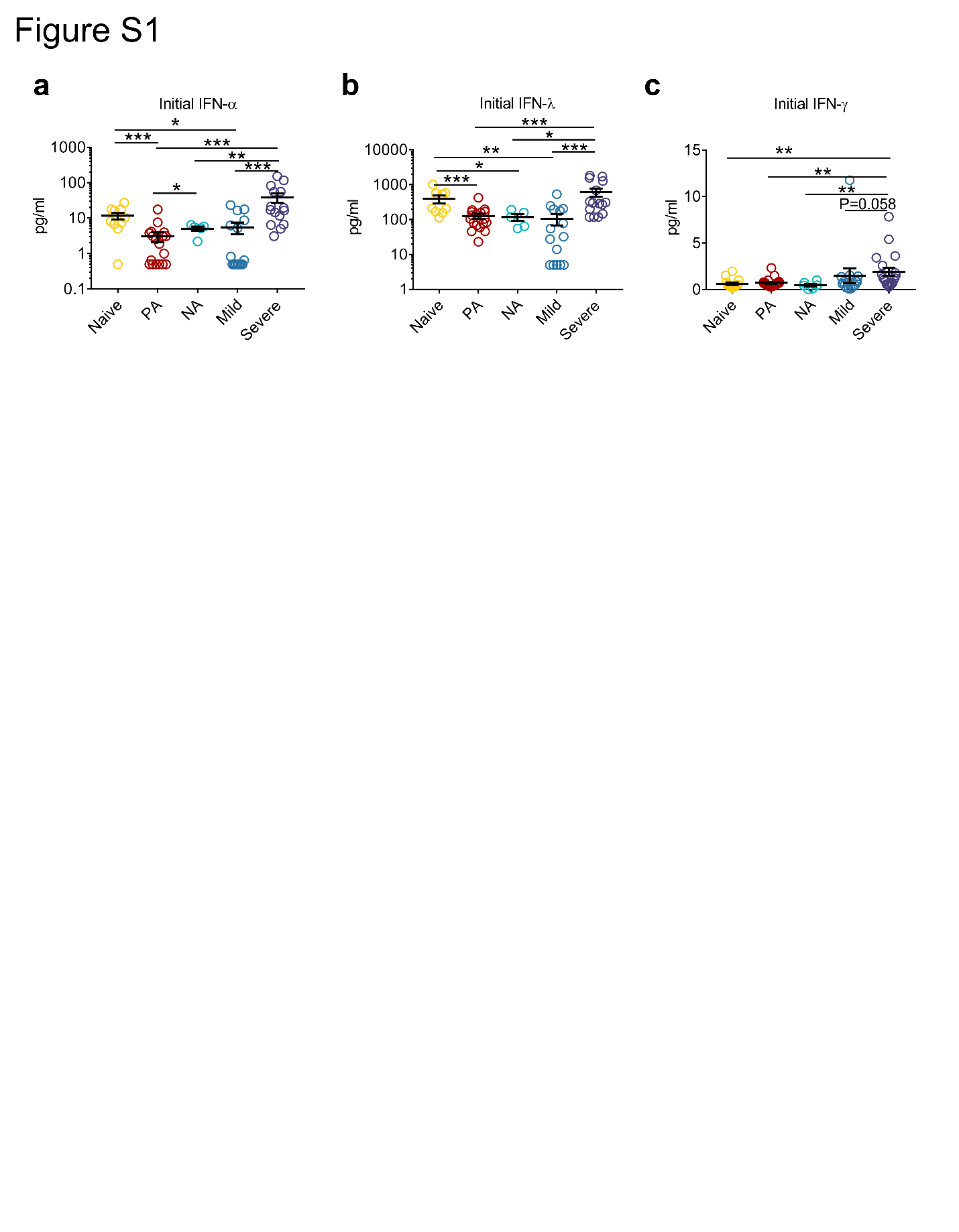


Figure. S1.

**Low initial interferon responses were induced in PA cases. a-c** Mann-Whitney test was used to compare the initial plasma values of IFN-α (**a**), IFN-λ (**b**) and IFN-γ (**c**) in each group at 1-2 wpo. * *P* < 0.05; ** *P* < 0.01; *** *P* < 0.001.


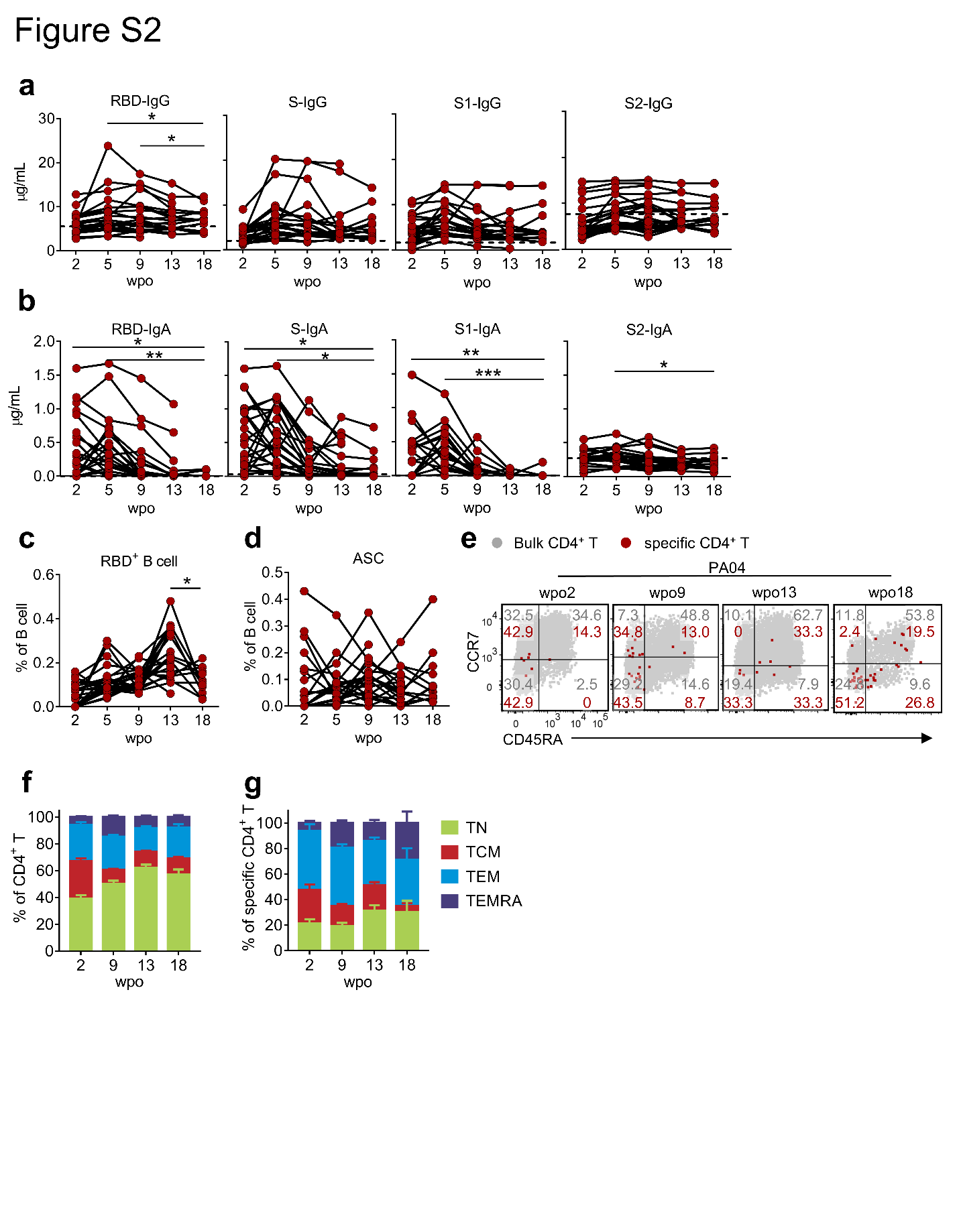


Figure. S2.

**Dynamics of immune responses in PA cases from 2 to 18 wpo. a, b** Dynamics of IgG (**a**) and IgA (**b**) levels against multiple structural proteins (RBD, S, S1, and S2) in the plasma are presented. Plasma samples from six healthy donors collected in 2017­-2018 were used to define the thresholds (mean + 3SD) indicated by dashed lines. **c, d** Frequencies of RBD^+^ B cells (**c**) and ASCs (**d**) in PA cases at various time points. **a-d** Wilcoxon matched-pairs signed rank test was used to compare data at different time points in the PA group. * *P* < 0.05; ** *P* < 0.01; *** *P* < 0.001. **e-g** Representative flow plots (**e**) and summary of memory subsets of total (**f**) and specific (**g**) CD4^+^ T cells in PA cases at 2, 9, 13, and 18 wpo are presented.


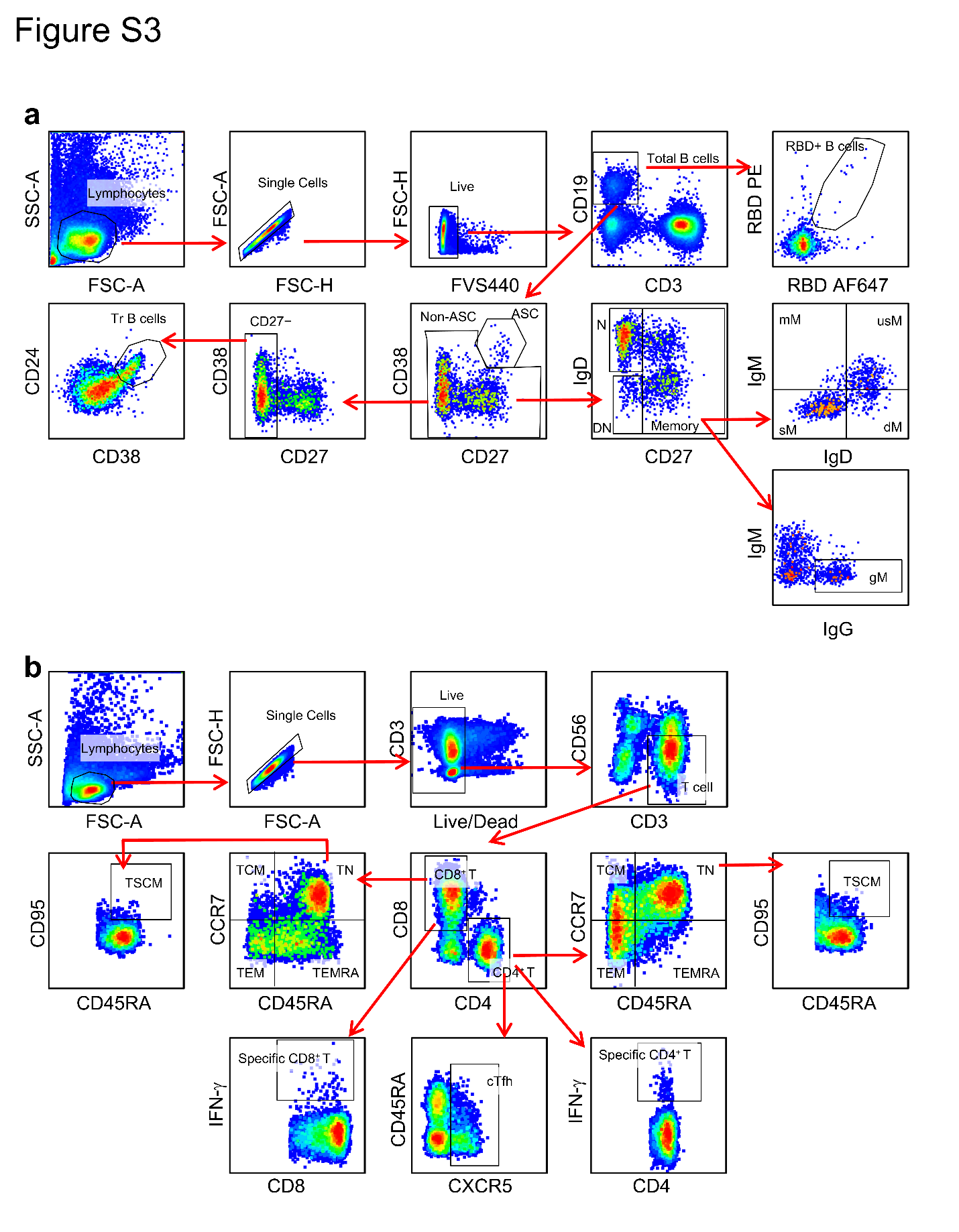


Figure. S3.

**Flow cytometric gating strategies.** **a, b** Flow gating strategies for the identification and quantification of ASCs, SARS-CoV-2-RBD^+^ B cells (**a**), and SARS-CoV-2-specific CD4^+^ and CD8^+^ T cells (**b**).


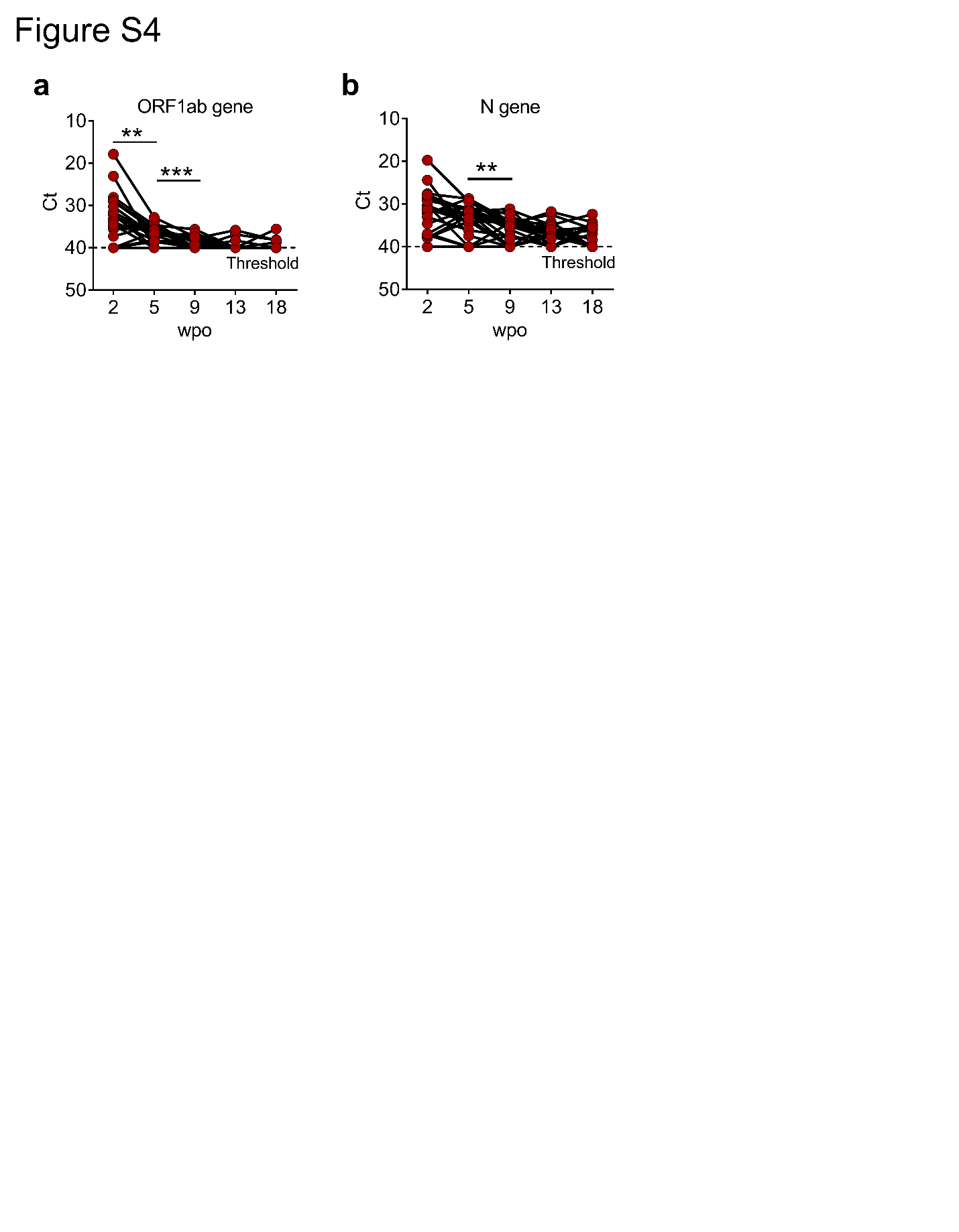


Figure. S4.

**Dynamics of viral loads in PA cases from 2 to 18 wpo.** **a, b** Viral loads in PA cases at various time points are shown, represented as Ct values of *ORF1ab* (**a**) and *N* genes (**b**). Dashed lines represent the threshold, and a Ct value of < 40 indicates the presence of SARS-CoV-2 nucleic acids in the sample.


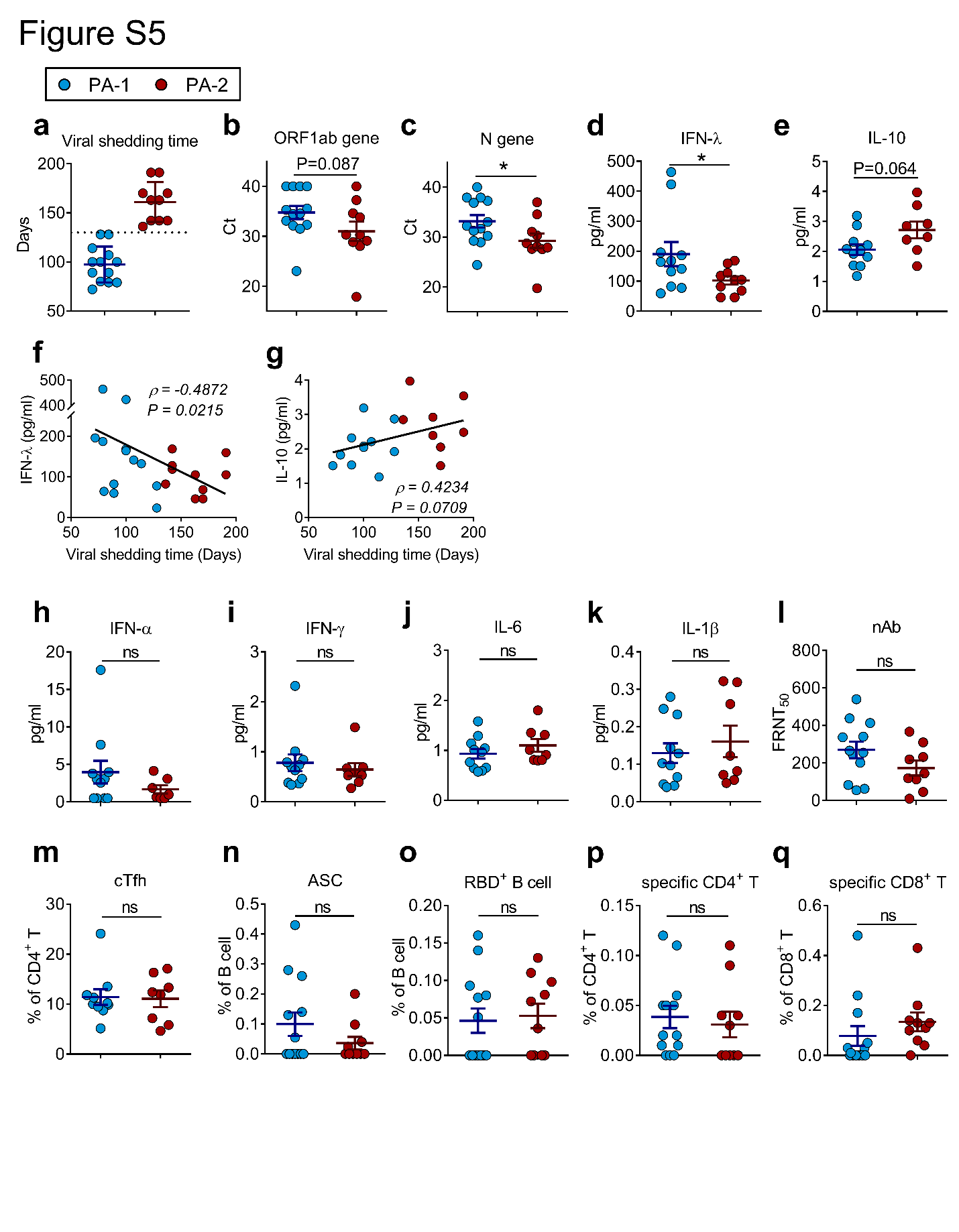


Figure. S5.

**In the PA group, delayed viral clearance was associated with initially lower IFN- and higher IL-10 levels.** **a** The PA cases were divided into two subgroups according to the viral shedding time: PA-1 (n=13, viral shedding time <130 days); PA-2 (n=10, viral shedding time >130 days). **b-e** Mann-Whitney test was used to compare initial values of Ct values of *ORF1ab* (**b**) and *N* genes (**c**), levels of IFN-λ (**d**) and IL-10 (**e**) in the two subgroups at 2 wpo. * *P* < 0.05. **f, g** Spearman correlation analysis was performed to analyze the correlation between viral shedding time and initial values of IFN-λ (**f**) and IL-10 (**g**). **h-q** Mann-Whitney test was used to compare levels of IFN-α (**h**), IFN-γ (**i**), IL-6 (**j**), IL-1β (**k**) and nAb (**l**) and frequencies of cTfhs (**m**), ASCs (**n**), RBD^+^ B cells (**o**), specific CD4^+^ T cells (**p**) and specific CD8^+^ T cells (**q**) in the two subgroups at 2 wpo. ns: *P* ≥ 0.05.


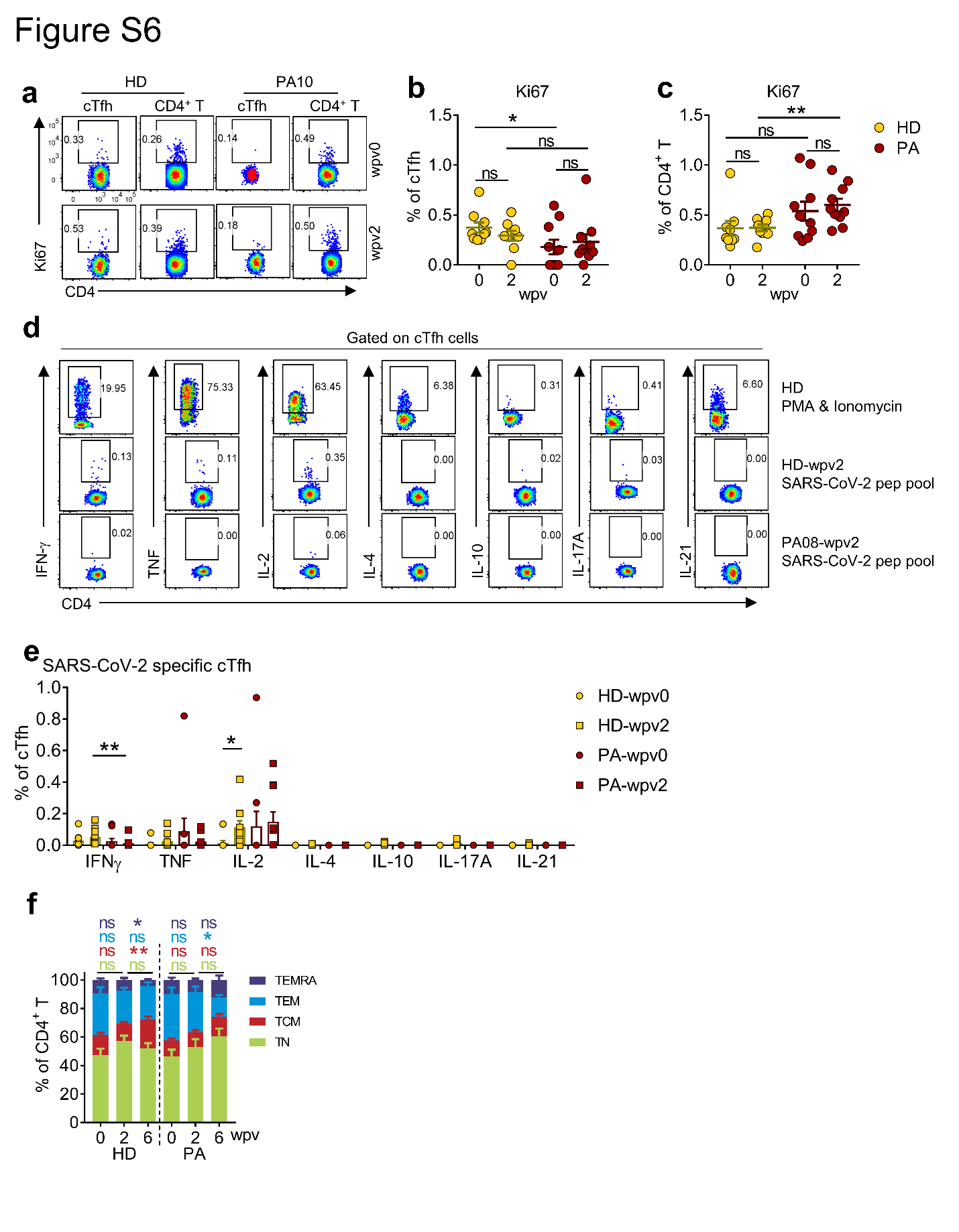


Figure. S6.

**PA cases exhibited unique immune response characteristics post-vaccination with Ad5-nCoV.** **a-c** Representative flow plots (**a**) and summary of Ki67 expression in cTfh (**b**) and CD4^+^ T cells (**c**) at 0 and 2 wpv in PA and HD individuals. **d, e** Representative flow plots (**d**) and summary of frequencies of SARS-CoV-2-specific cTfhs expressing different cytokines at 0 and 2 wpv in the PA and HD groups are presented (**e**). **e** The presented values from peptide-stimulated cells were subtracted by values from unstimulated cells. **f** Memory subset composition of CD4^+^ T cells at 0 and 2 wpv in the two groups is presented. Wilcoxon matched-pairs signed rank test was used to compare data at different time points in the same group. Mann−Whitney U test was used to compare data of two groups at the same time point. Data are presented as the mean ± SEM.

Table S1.

Cohort demographics.

| **PT ID** | **Group** | **Number of cases** | **Sex** | **Age (y)** | **Age (y) *Median (IQR)*** | **Virus Shedding Time (days)** | **Virus Shedding Time (days) *Median (IQR)*** | **Comorbidity** | **Comorbidity rate (%)** |
| --- | --- | --- | --- | --- | --- | --- | --- | --- | --- |
| PA01 | Persistent asymptomatic (PA) | 23 | Male | 25 | 29 (26−32) | 170 | 128 (89−163) | / | 0 |
| PA02 |  |  | Male | 24 |  | 142 |  | / |  |
| PA03 |  |  | Male | 34 |  | 142 |  | / |  |
| PA04 |  |  | Male | 26 |  | 136 |  | / |  |
| PA05 |  |  | Male | 30 |  | 163 |  | / |  |
| PA06 |  |  | Male | 29 |  | 163 |  | / |  |
| PA07 |  |  | Male | 32 |  | 170 |  | / |  |
| PA08 |  |  | Male | 27 |  | 142 |  | / |  |
| PA09 |  |  | Male | 26 |  | 191 |  | / |  |
| PA10 |  |  | Male | 26 |  | 191 |  | / |  |
| PA11 |  |  | Male | 31 |  | 100 |  | / |  |
| PA12 |  |  | Male | 29 |  | 79 |  | / |  |
| PA13 |  |  | Male | 32 |  | 89 |  | / |  |
| PA14 |  |  | Male | 32 |  | 128 |  | / |  |
| PA15 |  |  | Male | 31 |  | 100 |  | / |  |
| PA16 |  |  | Male | 25 |  | 107 |  | / |  |
| PA17 |  |  | Male | 27 |  | 128 |  | / |  |
| PA18 |  |  | Male | 32 |  | 89 |  | / |  |
| PA19 |  |  | Male | 35 |  | 100 |  | / |  |
| PA20 |  |  | Male | 31 |  | 114 |  | / |  |
| PA21 |  |  | Male | 33 |  | 72 |  | / |  |
| PA22 |  |  | Male | 25 |  | 79 |  | / |  |
| PA23 |  |  | Male | 27 |  | 80 |  | / |  |
| NA01 | Non-persistent asymptomatic (NA) | 6 | Male | 37 | 34 (26−56)^#^ | 9 | 11 (9−15)* | / | 50.0 |
| NA02 |  |  | Male | 30 |  | 9 |  | / |  |
| NA03 |  |  | Male | 24 |  | 9 |  | / |  |
| NA04 |  |  | Male | 65 |  | 15 |  | Chronic pharyngitis |  |
| NA05 |  |  | Female | 62 |  | 13 |  | T2D, Hypertension |  |
| NA06 |  |  | Female | 9 |  | 17 |  | Mild anemia |  |
| M01 | Mild | 19 | Male | 56 | 50 (28−57)* | 23 | 23 (20−27)* | / | 26.3 |
| M02 |  |  | Male | 24 |  | 23 |  | / |  |
| M03 |  |  | Male | 35 |  | 28 |  | / |  |
| M04 |  |  | Male | 48 |  | 28 |  | / |  |
| M05 |  |  | Male | 69 |  | 24 |  | / |  |
| M06 |  |  | Male | 77 |  | 27 |  | / |  |
| M07 |  |  | Male | 24 |  | 21 |  | / |  |
| M08 |  |  | Male | 28 |  | 41 |  | / |  |
| M09 |  |  | Male | 52 |  | 13 |  | Renal calculus |  |
| M10 |  |  | Male | 50 |  | 16 |  | Hypertension |  |
| M11 |  |  | Male | 45 |  | 23 |  | T2D |  |
| M12 |  |  | Female | 27 |  | 29 |  | / |  |
| M13 |  |  | Female | 54 |  | 24 |  | / |  |
| M14 |  |  | Female | 23 |  | 22 |  | / |  |
| M15 |  |  | Female | 25 |  | 15 |  | / |  |
| M16 |  |  | Female | 57 |  | 25 |  | / |  |
| M17 |  |  | Female | 50 |  | 18 |  | / |  |
| M18 |  |  | Female | 64 |  | 27 |  | Hypertension |  |
| M19 |  |  | Female | 77 |  | 10 |  | Aortosclerosis |  |
| S01 | Severe | 25 | Male | 50 | 58 (49−66)* | 61 | 55 (40−63)* | Cooley's anemia | 64.0 |
| S02 |  |  | Male | 63 |  | 40 |  | Hypertension, Arthrolithiasis |  |
| S03 |  |  | Male | 48 |  | 27 |  | Hypertension |  |
| S04 |  |  | Male | 49 |  | 53 |  | / |  |
| S05 |  |  | Male | 49 |  | 74 |  | T2D, COPD |  |
| S06 |  |  | Male | 53 |  | 95 |  | T2D, Coronary disease |  |
| S07 |  |  | Male | 42 |  | 79 |  | / |  |
| S08 |  |  | Male | 58 |  | 61 |  | / |  |
| S09 |  |  | Male | 26 |  | 70 |  | Hypohepatia |  |
| S10 |  |  | Male | 41 |  | 45 |  | Hypertension, Allergic rhinitis |  |
| S11 |  |  | Male | 56 |  | 38 |  | / |  |
| S12 |  |  | Male | 64 |  | 58 |  | T2D, COPD |  |
| S13 |  |  | Male | 61 |  | 55 |  | Hypertension, CH-B, SAS |  |
| S14 |  |  | Male | 72 |  | 69 |  | T2D, COPD, Hypertension |  |
| S15 |  |  | Male | 79 |  | 63 |  | T2D, Hypertension |  |
| S16 |  |  | Male | 66 |  | 52 |  | / |  |
| S17 |  |  | Male | 68 |  | 37 |  | Hypertension, Coronary disease, COPD |  |
| S18 |  |  | Female | 72 |  | 53 |  | Brain tumor |  |
| S19 |  |  | Female | 55 |  | 67 |  | / |  |
| S20 |  |  | Female | 65 |  | 57 |  | T2D, Hypertension, Cerebral infarction |  |
| S21 |  |  | Female | 39 |  | 48 |  | / |  |
| S22 |  |  | Female | 73 |  | 19 |  | / |  |
| S23 |  |  | Female | 60 |  | 40 |  | / |  |
| S24 |  |  | Female | 82 |  | 58 |  | Hypertension, Arthrolithiasis |  |
| S25 |  |  | Female | 56 |  | 27 |  | Hypertension |  |
| *#*: *P≥0.05, **: *P<*0.05*.* Each group was compared with the PA group. /: none; T2D: Type II diabetes; COPD: chronic obstructive pulmonary disease; CH-B: Chronic viral hepatitis B; SAS: Sleep Apnea Syndrome. | | | | | | | | | |

Table S2.

Vaccination information.

| **ID** | **Group** | **Sex** | **Age (y)** | **Age (y) *Median (IQR)*** | **1^st^ dose of vaccine** | **2^nd^ dose of vaccine** | **3^rd^ dose of vaccine** | **Time interval between 2^nd^ and 3^rd^ dose of vaccine** |
| --- | --- | --- | --- | --- | --- | --- | --- | --- |
| PA01 | PA | Male | 25 | 27 (26−30) | Convidecia (Inhalation) | N.A. | N.A. | N.A. |
| PA02 |  | Male | 24 |  | Convidecia (Inhalation) |  |  |  |
| PA03 |  | Male | 34 |  | Convidecia (Inhalation) |  |  |  |
| PA04 |  | Male | 26 |  | Convidecia (Inhalation) |  |  |  |
| PA05 |  | Male | 30 |  | Convidecia (Inhalation) |  |  |  |
| PA06 |  | Male | 29 |  | Convidecia (Inhalation) |  |  |  |
| PA07 |  | Male | 32 |  | Convidecia (Inhalation) |  |  |  |
| PA08 |  | Male | 27 |  | Convidecia (Inhalation) |  |  |  |
| PA09 |  | Male | 26 |  | Convidecia (Inhalation) |  |  |  |
| PA10 |  | Male | 26 |  | Convidecia (intramuscular) |  |  |  |
| HD01 | HD | Male | 23 | 26 (23-35) | Coronavac | Coronavac | Convidecia (Inhalation) | 8 months |
| HD02 |  | Male | 22 |  | Coronavac | BBIBP-CorV | Convidecia (Inhalation) | 6 months |
| HD03 |  | Male | 43 |  | Coronavac | Coronavac | Convidecia (Inhalation) | 6 months |
| HD04 |  | Male | 43 |  | Coronavac | Coronavac | Convidecia (Inhalation) | 7 months |
| HD05 |  | Male | 22 |  | BBIBP-CorV | Coronavac | Convidecia (Inhalation) | 7 months |
| HD06 |  | Male | 35 |  | Coronavac | Coronavac | Convidecia (Inhalation) | 7 months |
| HD07 |  | Female | 23 |  | Coronavac | Coronavac | Convidecia (Inhalation) | 11 months |
| HD08 |  | Female | 25 |  | BBIBP-CorV | BBIBP-CorV | Convidecia (Inhalation) | 9 months |
| HD09 |  | Female | 26 |  | BBIBP-CorV | Coronavac | Convidecia (Inhalation) | 5 months |
| HD10 |  | Female | 23 |  | BBIBP-CorV | BBIBP-CorV | Convidecia (Inhalation) | 10 months |
| HD11 |  | Female | 35 |  | BBIBP-CorV | BBIBP-CorV | Convidecia (Inhalation) | 9 months |
| HD12 |  | Female | 27 |  | Coronavac | Coronavac | Convidecia (Inhalation) | 6 months |
| HD13 |  | Female | 34 |  | BBIBP-CorV | BBIBP-CorV | Convidecia (Inhalation) | 8 months |
| HD14 |  | Female | 44 |  | Coronavac | Coronavac | Convidecia (Inhalation) | 6 months |
| HD15 |  | Female | 26 |  | Coronavac | Coronavac | Convidecia (Inhalation) | 6 months |
| N.A., not applied. | | | | | | | | |
